# P-1455. Enhancing vaccination through a pharmacist-directed transitions of care service in a safety-net hospital

**DOI:** 10.1093/ofid/ofaf695.1641

**Published:** 2026-01-11

**Authors:** Marissa Cavaretta, Rhonda Abouelela, Nicholas Ferraro, Jason C Gallagher

**Affiliations:** Temple University, Philadelphia, Pennsylvania; St Peter's University Hospital, New Brunswick, New Jersey; Temple University Hospital, Philadelphia, Pennsylvania; Temple University, Philadelphia, Pennsylvania

## Abstract

**Background:**

Pharmacist-directed vaccination programs in the outpatient community setting improve vaccination rates and increase access to care. This project explored whether a pharmacist-direct transitions of care (TOC) consult service could identify and rectify gaps in vaccination in an at-risk population.

**Figure 1:**
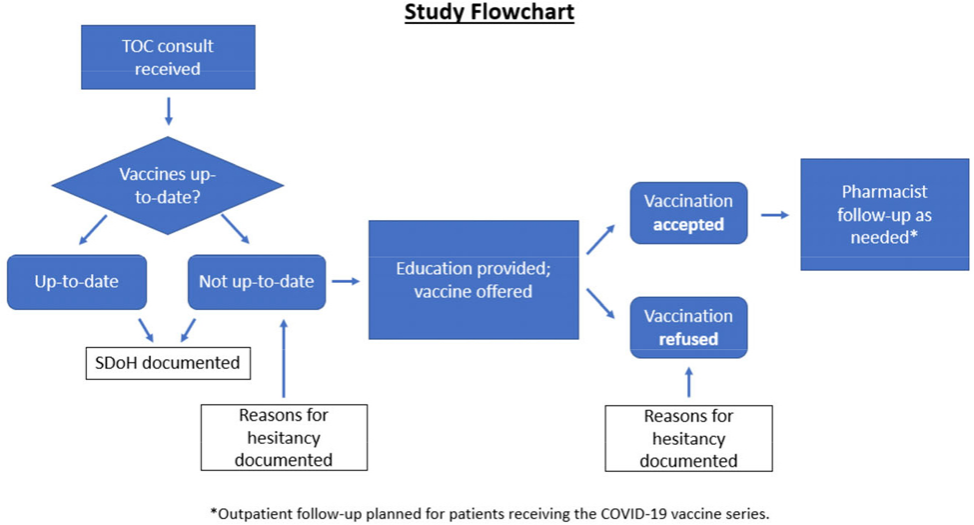

**Figure:**
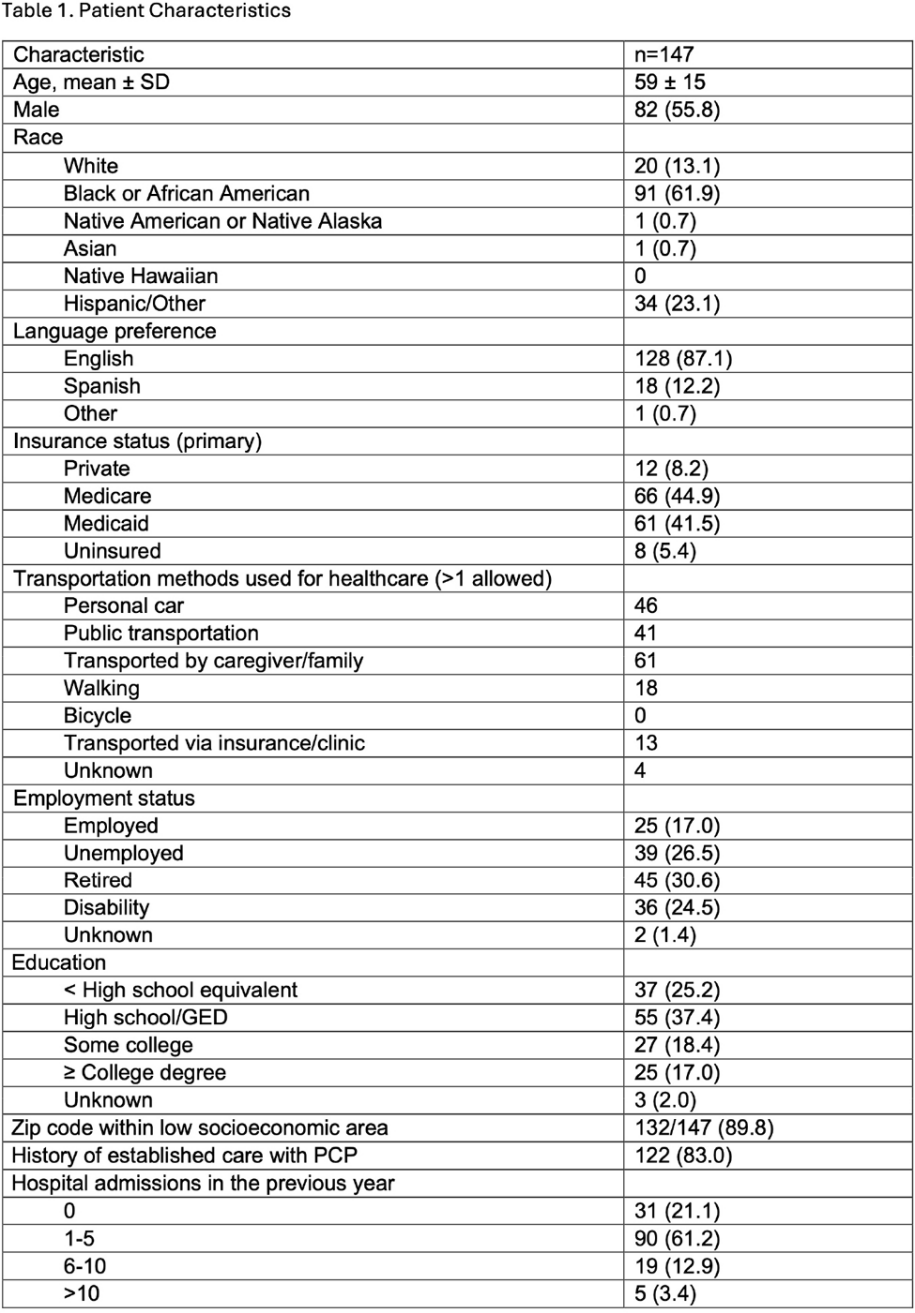

**Methods:**

This was a quality improvement initiative evaluating the impact of inpatient pharmacist interventions on vaccine rates. Pharmacists on the TOC consult service incorporated a vaccine review into the service workflow to identify adult patients indicated for influenza, pneumococcal, and COVID-19 vaccinations. Included patients were adults 18 years of age or older who were referred to the TOC service. Pregnant women, prisoners, patient directed discharges, and patients with contraindications to receiving vaccines were excluded. Patients on the service were screened for vaccine status using a standardized workflow (Figure 1). All patients were interviewed to obtain information relating to Social Determinants of Health (SDoH) and vaccine status. For patients not up-to-date, reasons for vaccine hesitancy were collected. Interventions were made to providers of non-up-to-date patients who agreed to receive immunizations. The primary objective was to assess if an inpatient pharmacist-directed TOC service increases vaccination rates. Descriptive statistics were used to summarize the data.

**Figure:**
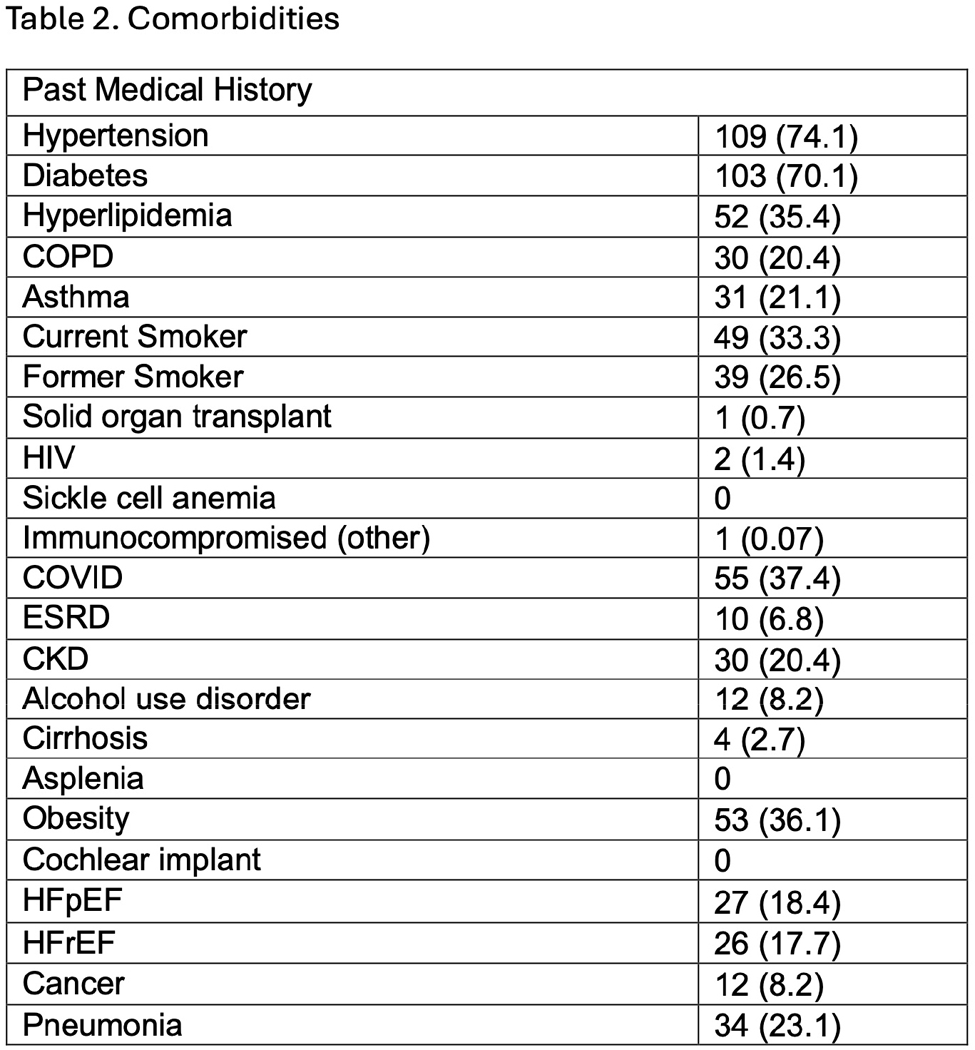

**Figure:**
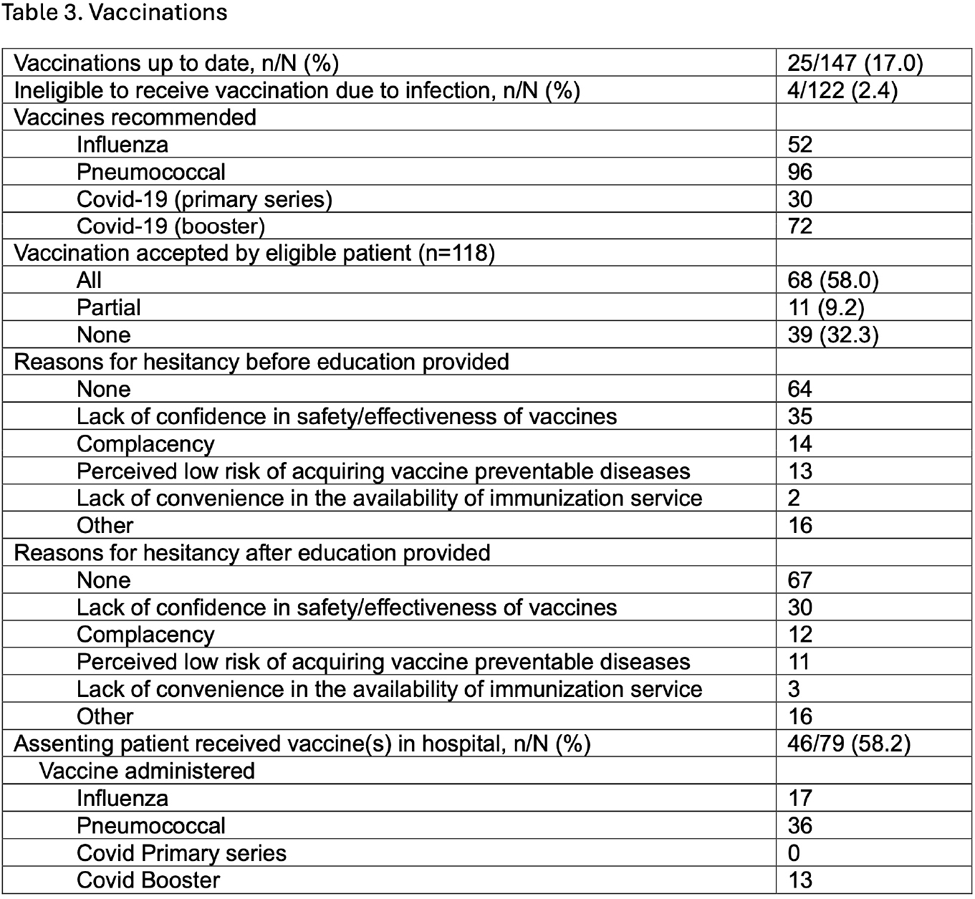

**Results:**

Results are stated in tables 1-3.

**Conclusion:**

A significant majority of patients had incomplete vaccination histories, with missed or delayed doses identified. Most patients agreed to be vaccinated during their inpatient stay, leading to timely pharmacist-initiated interventions. In a population with SDoH that may influence vaccination rates, incorporating vaccination screenings and recommendations into a pharmacist-directed service resulted in an increased number of vaccinations administered.

**Disclosures:**

Marissa Cavaretta, PharmD, Merck & Co: Grant/Research Support Jason C. Gallagher, PharmD, AbbVie: Advisor/Consultant|AbbVie: Honoraria|GSK: Advisor/Consultant|Invivyd: Advisor/Consultant|Merck & Co: Grant/Research Support|Metheal: Advisor/Consultant|Novavax: Advisor/Consultant|Shionogi: Advisor/Consultant|Shionogi: Honoraria

